# Prevalence of HIV infection and bacteriologically confirmed tuberculosis among individuals found at bars in Kampala slums, Uganda

**DOI:** 10.1038/s41598-020-70472-6

**Published:** 2020-08-10

**Authors:** Joseph Baruch Baluku, Godwin Anguzu, Sylvia Nassozi, Febronius Babirye, Sharon Namiiro, Robert Buyungo, Mike Sempiira, Amir Wasswa, Rose Mulwana, Samuel Ntambi, William Worodria, Irene Andia-Biraro

**Affiliations:** 1 Department of Internal Medicine, Makerere University College of Health Sciences, PO Box 7072, Kampala, Uganda; 2 Mulago National Referral Hospital, P.O. Box 7051, Pulmonology Division, Uganda; 3 Mildmay Uganda, P.O. Box 24985, Kampala, Uganda; 4 MRC/UVRI & LSHTM Uganda Research Unit, P.O. Box 49, Entebbe, Uganda; 5 Makerere University Infectious Disease Institute, P.O. Box, 22418 Kampala, Uganda; 6 Makerere University Lung Institute, P.O. Box 7749, Kampala, Uganda

**Keywords:** HIV infections, Tuberculosis

## Abstract

Individuals found at bars in slums have several risk factors for HIV and tuberculosis (TB). To determine the prevalence of HIV and TB among individuals found at bars in slums of Kampala, Uganda, we enrolled adults found at bars that provided written informed consent. Individuals with alcohol intoxication were excluded. We performed HIV testing using immunochromatographic antibody tests (Alere Determine HIV-1/2 and Chembio HIV 1/2 STAT-PAK). TB was confirmed using the Xpert MTB/RIF Ultra assay, performed on single spot sputum samples. We enrolled 272 participants from 42 bars in 5 slums. The prevalence of HIV and TB was 11.4% (95% CI 8.1–15.8) and 15 (95% CI 6–39) per 1,000 population respectively. Predictors of HIV were female sex (aOR 5.87, 95% CI 2.05–16.83), current cigarette smoking (aOR 3.23, 95% CI 1.02–10.26), history of TB treatment (aOR 10.19, 95% CI 3.17–32.82) and CAGE scores of 2–3 (aOR 3.90, 95% CI 1.11–13.70) and 4 (aOR 4.77, 95% CI 1.07–21.35). The prevalence of HIV and TB was twice and four times the national averages respectively. These findings highlight the need for concurrent programmatic screening for both HIV and TB among high risk populations in slums.

## Introduction

HIV and tuberculosis (TB) interact at an epidemiological, clinical, cellular, and molecular level to create a co-epidemic^1^. In 2018, HIV contributed 251,000 of the 1.2 million TB deaths while TB was the leading cause of death among HIV positive individuals^2,3^. Notwithstanding, an estimated 3 million TB cases were missed in 2018 and only 79% of HIV-positive individuals knew their HIV status^2,3^. To increase the detection of TB and HIV, it is important to target high risk and vulnerable populations through active community based screening strategies^4–6^.

Slum dwellers have a higher risk for HIV, TB and HIV/TB co-infection than the national averages^7,8^. However, slum dwellers are less likely to utilise health services for HIV and TB diagnosis and have a low level of knowledge regarding prevention strategies for either disease^9,10^. The low utilisation is partly attributed to the perceived poor quality of services at public facilities and thus high risk groups are not covered by facility based screening strategies^11,12^. Within slum settlements, bars and social drinking places carry the highest risk for TB transmission than other social gathering places such as churches, clinics, hospitals, taxis, community halls, schools, and supermarkets^13–16^. As such, bars and social alcohol drinking groups are avenues for TB transmission to bar customers, employees and neighbours, and they propagate outbreaks from an index case^17–21^. Moreover, alcohol consumption is an established risk factor for tuberculosis in a dose dependent fashion, and exacerbates TB infection by blunting CD4 and CD8 T-lymphocyte cellular responses^22,23^.

Similarly, there is a high prevalence of HIV in areas that have a high density of bars^24^. It is likely that the prevalence of HIV among bar customers, employees and neighbours of bars in slums is high due to the high risk sexual behaviour, low risk perception of sex with commercial sex workers and inconsistent condom use observed among individuals at bars in slums^21,25,26^. Further, individuals who attend bars are less likely to visit health facilities and new TB cases and HIV infections among customers, staff and residents at bars in slums are missed^12^. The prevalence of HIV and TB among individuals found at bars in slum settings is not widely reported because studies in this setting have focused on each disease in isolation.

The aim of this study was to determine the prevalence of HIV and TB and predictors of HIV infection among individuals found at bars in Kampala slums in Uganda.

## Materials and Methods

### Study population and setting

Using a cross sectional study design, we enrolled participants from the 5 divisions of Kampala city, the capital city of Uganda, in December of 2019. Kampala city is sub-divided into 5 administrative divisions: Kawempe, Lubaga, Makindye, Nakawa and Central divisions. A map supplied by the Kampala Capital City Authority designates 29 settlements as slums. We randomly selected one slum from each division of Kampala. Bwaise, Kamwokya, Nalukolongo, Kibuli and Naguru Go-down slums were selected from Kawempe, Central, Lubaga, Makindye and Nakawa divisions respectively. At each slum settlement, we identified bars by snowball sampling method with the aid of a village health team leader or a local council chairperson due to lack of organised housing and registration status of bars in slums. The lack of official licensing of the bars and their operation outside the legally recommended hours makes individuals attending these places a “hard-to-reach” and “hidden” population that justifies the use of the snowball sampling method^27^. A bar was defined as any place from which alcoholic beverages were sold and consumed at the premises. At each bar, study participants were sampled and enrolled consecutively until the desired sample size for the given slum was achieved. The study inclusion criteria were any adult (≥ 18 years) who was found at a bar during the study visit and provided written informed consent. We excluded potential participants with alcohol intoxication as this would impair ability to provide informed consent. Alcohol intoxication was defined as Hack’s impairment index of ≥ 0.6^28^.

### Study measurements

We administered a pre-tested questionnaire that sought for socio-demographic data, history of alcohol and cigarette use, history of TB exposure and treatment and past medical history. The socio-economic status of participants was measured by the number of durable household items using the equity tool available at: https://www.equitytool.org/the-equity-tool-2/^29^. Using possession of common household items as a measure of household wealth is used in national demography surveys in Uganda^30^. The socioeconomic status was divided into 3 tertiles: rich, middle class and poor after principal component analysis. We used the CAGE questionnaire to assess the level of alcohol consumption. The CAGE questionnaire is a set of 4 simple questions: “Have you ever: (1) felt the need to cut down your drinking; (2) felt annoyed by criticism of your drinking; (3) had guilty feelings about drinking; and (4) taken a morning eye opener?”^31^. A score of 2–3 is indicative of a high index of suspicion for alcohol abuse while a score of 4 is diagnostic of alcohol dependence^32^. A study nurse drew 1 ml of blood following standard procedures^33^. We performed HIV testing on whole blood samples using a rapid immunochromatographic antibody test (Alere Determine HIV-1/2) and sequentially confirmed positive samples with another test (Chembio HIV 1/2 STAT-PAK) according to the Uganda ministry of health HIV testing guidelines^34^. Participants also provided single spot sputum samples that were tested using the Xpert MTB/RIF Ultra assay for bacteriological confirmation of TB at Mulago Hospital tuberculosis unit laboratory according to World Health Organisation Xpert MTB/RIF user guidelines^35^. Spot sputum samples are equally sensitive as early morning sputum samples for TB diagnosis^36^. Twenty one participants were unable to expectorate sputum spontaneously. For these, we induced sputum by nebulising 0.45% hypertonic saline using an ultrasonic nebuliser following normal post bronchodilator spirometry findings according to standard procedures^37^. To ensure participant privacy and confidentiality, we delivered pre- and post–HIV testing counselling in a designated housing unit in the slum. Newly diagnosed HIV-positive and bacteriologically confirmed TB participants were linked to Makerere University Joint AIDS Program and Mulago Hospital tuberculosis treatment unit for HIV and TB care respectively.

### Sample size estimation and statistical analysis

Using the prevalence of sputum positive TB among Kampala slum dwellers with a chronic cough of 18%^38^, the sample size was estimated to be 283 considering a 95% confidence interval and possible incomplete data rate of 20%. Similarly, a sample size of 178 individuals was deemed adequate for estimating the prevalence of HIV considering a 10.3% prevalence of HIV among youths in slums of Kampala^39^. The sample size was distributed across the 5 sampled slum settlements proportional to population size of the division using data from the Uganda demographic survey^30^. Data were entered in EpiData 3.1 and analysed with Stata 15 (StataCorp, College Station, TX, USA). The prevalence of HIV was estimated as the proportion of participants with a positive HIV test to the total number of study participants, expressed as a percentage. Similarly, the prevalence of bacteriologically confirmed TB was estimated as the proportion of participants with a positive Xpert MTB/RIF Ultra assay to the total participants, expressed per 1,000 population. As a secondary objective, we performed logistic regression analysis to determine predictors of HIV infection. At univariable analysis, we examined the associations between categorical and continuous variables and HIV infection using chi-square tests and Rank sum test respectively. Prior to multivariate analysis, we assessed the individual associations between independent variables and HIV infection using individual simple logistic regression analysis. Variables that showed associations with a cut off threshold of *p* value < 0.2 and didn’t have sparse data in categories were considered at the multivariable analysis. At multivariable analysis, we used a multivariable logistic regression model to obtain factors that independently predicted HIV infection at *p* < 0.05 and a 95% confidence interval.

### Ethical approvals and consent to participate

Study participants provided written informed consent to participate in the study. The study was approved by The AIDS Support Organisation Research and Ethics committee (ref number: TASOREC/044/19-UG-REC-009) and the Uganda National Council of Science and Technology (ref: HS459ES). All methods and experimental protocols were carried out in accordance to the declaration of Helsinki.

## Results

### Participant enrolment

We screened 342 potential participants found at bars in Kampala slums. Of these, 15 were < 18 years of age, 17 had alcohol intoxication and 38 withdrew consent.

### Participant characteristics

We enrolled 272 participants from 42 bars, of whom 170 (62.5%) were bar customers, 30 (11.0%) were bar owners, 30 (11.0%) were bar staff while 42 (15.4%) were merely found at the premises. Fourteen (5.2%), 58 (21.3%), 65 (23.9%), 71 (26.1%) and 62 (22.8%) participants were from Kamwokya, Bwaise, Nalukolongo, Kibuli and Naguru Go-down slum settlements respectively. There were 196 (72.1%) males and 251 (92.3%) participants were residents of the slum settlement they were found at with median (interquartile range, IQR) duration of residence of 8 (3–17) years. The median (IQR) age of study participants was 32 (27–38) years. The median (IQR) number of days participants had visited the bar in the preceding week was 7 (3–7) days. Other characteristics of the study participants are shown in Table 1.

**Table 1:** Characteristics of study participants.

Participant characteristic	Frequency (N = 272)	Percentage (%)
**Education level**		
None	23	8.4
Primary	85	31.2
Secondary	136	50.0
Tertiary	28	10.3
**Employment status**		
Employed	246	90.4
Unemployed	26	9.6
**Socioeconomic status**		
Rich	91	33.5
Middle class	93	34.2
Poor	88	32.3
**History of smoking**		
Never	159	58.5
Past smoker (≥ 6 months ago)	36	13.2
Current smoker (< 6 months)	77	28.3
**Alcohol use**		
Never used	43	15.8
Past alcohol use (≥ 6 months)	43	15.8
Current alcohol use (< 6 months)	186	68.4
**Tuberculosis (TB) symptoms**		
Cough	98	36.0
Weight loss	68	25.0
Night sweats	65	23.9
Chest pain	62	22.8
Anorexia	57	21.0
Fever	52	19.1
Difficulty in breathing	43	15.8
Haemoptysis	7	2.6
**TB treatment history**		
Currently on TB treatment	3	1.1
Previous history of TB treatment	23	8.5
**History of TB contact**		
Contact with known TB case	62	22.8
Contact with person with a chronic cough	43	15.8
**History of comorbidities**		
Hypertension	12	4.4
Asthma	8	2.9
Chronic liver disease	4	1.5
Cancer	3	1.1
Diabetes	2	0.7
Cardiac disease	2	0.7
Psychiatric illness	2	0.7
Chronic kidney disease	1	0.4
**CAGE**		
0–1	102	37.5
2–3	121	44.5
4	38	14.0

### Prevalence of HIV infection and bacteriologically confirmed TB among individuals found at bars

The prevalence of HIV was 11.4% (31/272) (95% confidence interval (CI): 8.1–15.8) and 16 (51.6%) individuals were newly diagnosed with HIV. The prevalence of HIV was statistically different across the slum settlements, that is, 25.9% (15/58) in Bwaise, 21.4% (3/14) in Kamwokya, 9.2% (6/65) in Nalukolongo, 7.0% (5/71) in Kibuli and 3.2% (2/62) in Naguru Go-down (*p* = 0.001). Among the study participants, 235 (82.7%) had ever tested for HIV. Of these, 15 (6.4%) reported to be known HIV positive individuals. Among those that self-reported to be HIV positive, 14 (93.3%) were experienced on antiretroviral therapy (ART) while 6 (40.0%) reported to be taking cotrimoxazole prophylaxis. The proportion of HIV positive participants was higher among those who had never tested for HIV than those who had ever tested (14.9% vs. 10.7%, *p* < 0.001). The other characteristics of HIV positive individuals are shown in Table 2.

**Table 2:** Characteristics of HIV positive individuals found at bars in slums of Kampala.

Characteristic	HIV test result		*p* value^†^
	Negative N = 241 (%)	Positive N = 31 (%)	
**Age; median (interquartile range)**	32 (27–38)	31 (26–38)	0.942^a^
**Slum settlement**			0.001^1^
Kamwokya	11 (78.6)	3 (21.4)	
Bwaise	43 (74.1)	15 (25.9)	
Nalukolongo	59 (90.8)	6 (9.2)	
Kibuli	66 (93.0)	5 (7.0)	
Naguru	60 (96.8)	2 (3.2)	
**Respondent type**			0.089^1^
Bar owner	26 (86.7)	4 (13.3)	
Customer	150 (88.2)	20 (11.8)	
Staff	24 (80.0)	6 (20.0)	
Other	41 (97.6)	1 (2.4)	
**Sex**			
Male	180 (91.8)	16 (8.2)	0.007
Female	61 (80.3)	15 (19.7)	
**Education level**			
None	20 (87.0)	3 (13.0)	0.591^1^
Primary	75 (88.2)	10 (11.8)	
Secondary	119 (87.5)	17 (12.5)	
Tertiary	27 (96.4)	1 (3.6)	
**History of cigarette smoking**			0.009^1^
Never	148 (93.1)	11 (6.9)	
Past (> 6 months ago)	32 (88.9)	4 (11.1)	
Current (< 6 months ago)	61 (79.2)	16 (20.8)	
**Alcohol use**			0.045^1^
Never used	40 (93.0)	3 (7.0)	
Past (> 6 months ago)	42 (97.7)	1 (2.3)	
Current (< 6 months ago)	159 (85.5)	27 (14.5)	
**Resident of slum**			0.145^1^
Yes	220 (87.7)	31 (13.3)	
No	21 (100.0)	0	
**Currently on TB treatment**			0.035^1^
Yes	1 (33.3)	2 (66.7)	
No	240 (89.2)	29 (10.8)	
**History of TB treatment**			< 0.001
Yes	13 (56.5)	10 (43.5)	
No	228 (91.6)	21 (8.4)	
**History of contact**			0.160
None	151 (91.5)	14 (8.5)	
Contact with known TB case	55 (88.7)	7 (11.3)	
Contact with person with a chronic cough	35 (81.4)	8 (18.6)	
**Employment status**			1.000^1^
Employed	218 (88.6)	28 (11.4)	
Unemployed	23 (88.5)	3 (11.5)	
**Socioeconomic status**			0.587
Rich	83 (91.2)	8 (8.8)	
Middle	82 (88.2)	11 (11.8)	
Poor	76 (86.4)	12 (13.6)	
**Any comorbidity**			0.331^1^
No history of comorbidity	218 (87.9)	30 (12.1)	
History of comorbidity	23 (95.8)	1 (4.2)	
**HIV self-reported status**			< 0.001^1^
Unknown	40 (85.1)	7 (14.9)	
Negative	201 (95.7)	9 (4.3)	
Positive	0	15 (100.0)	
**CAGE**			0.012
0–1	96 (94.1)	6 (5.9)	
2–3	105 (86.8)	16 (13.2)	
4	29 (76.3)	9 (23.7)	
Number of days visited bar in preceding week (median) (n = 229)	7 (3–7)	7 (3–7)	0.596^a^

^†^*p* value is generated from Pearson’s chi-square test, ^1^*p* value is from Fisher’s exact test, ^a^p-value is from Ranksum test.

There were 4 participants with newly diagnosed bacteriologically confirmed TB, corresponding to a prevalence of 15 (95%CI: 6–39) per 1,000 population. One individual had HIV/TB co-infection corresponding to a prevalence of 4 per 1,000 population. Thirty (11.0%) participants reported either history of TB treatment, current TB treatment or had newly diagnosed bacteriologically confirmed TB.

### Predictors of HIV infection among individuals found at bars in slums in Kampala

In a multivariable logistic regression model, female sex (adjusted odds ratio (aOR) 5.87, 95% confidence interval (CI) 2.05–16.83), current cigarette smoking (aOR 3.23, 95% CI 1.02–10.26), history of TB treatment (aOR 10.19, 95% CI 3.17–32.82) and CAGE scores of 2–3 (aOR 3.90, 95% CI 1.11–13.70) and 4 (aOR 4.77, 95% CI 1.07–21.35) independently predicted HIV infection. The multivariable model is shown in Table 3.

**Table 3:** Multivariable logistic regression model for predictors of HIV infection among individuals found at bars in slums of Kampala.

Characteristics	Crude odds ratio (95% CI)	*p* value	Adjusted odds ratio (95% CI)	*p* value
**Slum settlement**				
Kamwokya	1		1	
Bwaise	1.28 (0.31–5.22)	0.731	0.87 (0.17–4.52)	0.867
Nalukolongo	0.37 (0.08–1.72)	0.206	0.82 (0.14–4.70)	0.823
Kibuli	0.28 (0.06–1.33)	0.109	0.44 (0.08–2.58)	0.366
Naguru	0.12 (0.02–0.82)	0.03	0.28 (0.04–2.30)	0.239
**Sex**				
Male	1		1	
Female	2.77 (1.29–5.93)	0.009	5.87 (2.05–16.83)	**0.001**
**History of cigarette smoking**				
Never	1		1	
Past smoker (> 6 months ago)	1.68 (0.50–5.62)	0.398	1.70 (0.40–7.13)	0.470
Current smoker (< 6 months)	3.53 (1.55–8.04)	0.003	3.23 (1.02–10.26)	**0.047**
**History of TB treatment**				
No	1		1	
Yes	8.33 (3.27–21.28)	< 0.001	10.19 (3.17–32.82)	** < 0.001**
**History of TB contact**				
None	1		1	
Contact with known TB case	1.37 (0.53–3.58)	0.517	0.64 (0.20–2.03)	0.447
Contact with person with a chronic cough	2.47 (0.96–6.33)	0.061	1.89 (0.59–6.10)	0.287
**Alcohol Cage**				
0–1	1		1	
2–3	2.72 (1.02–7.21)	0.074	3.90 (1.11–13.70)	**0.047**
4	5.53 (1.82–16.82)	0.005	4.77 (1.07–21.35)	**0.041**

### Characteristics of Bacteriologically confirmed TB individuals found at bars in slums of Kampala

Of the 4 participants with bacteriologically confirmed TB, 3 were customers while 1 was a bar owner. Two were from Nalukolongo, 1 from Kibuli and 1 was from Naguru Go-down slum settlements. Also, of the bacteriologically confirmed TB individuals, 3 (75%) were male, 4 (100%) were slum residents, 3 (75%) reported current alcohol use, 3 (75%) reported history of a cough and 3 (75%) had history of contact with a known TB case.

## Discussion

The aim of this study was to determine the prevalence of HIV and bacteriologically confirmed TB among individuals found at bars in slums of Kampala. We further analysed for predictors of HIV infection in this population. We found the prevalence of HIV to be twice the national average of 5.7% among adults^40^. The prevalence of HIV significantly differed by slum settlement and was higher among participants who had never tested for HIV. Female sex, history of tuberculosis treatment, current history of cigarette smoking and CAGE scores of 2–3 and 4 were independent predictors of HIV infection. The prevalence of bacteriologically confirmed TB among individuals found at bars in Kampala slums was 4 times the national estimate of 4 per 1,000 population^41^. Our study findings show a high prevalence of HIV and TB among individuals found at bars in slums. Moreover, all TB patients and more than half of the HIV infections were newly diagnosed. These data indicate that many new HIV infections and TB cases are missed, and programmatic screening for the two infections is warranted among customers, staff, bar owners and other individuals found at bars in slums. The geospatial variation in the prevalence of HIV within the same city suggests a need for slum specific HIV prevention strategies^42^.

Similar to our findings, Lama et al*.* (2016), found the prevalence of HIV among bar patrons to be 12.7% in the capital of Botswana and 19% of first time testers were HIV positive^43^. In Tanzania, the prevalence of HIV was 19.0% among female bar and hotel workers which is comparable to our finding of 19.7% among females^44^. The prevalence of HIV is known to be higher among women than men in sub-Saharan Africa owing to different responses to socio-demographic risk factors, sexual behaviours and HIV/AIDS awareness^45,46^. However, we suspect that the high HIV risk observed among women found at bars in our study is attributed to commercial sex work engaged in by women at bars in Kampala slums as reported by Mbonye et al*.* (2013)^47^. Moreover, the high prevalence of HIV observed in Bwaise slum is consistent with a higher density of female commercial sex workers and men who have sex with men observed in Kawempe division of Kampala^48^. A further evaluation of the transmission dynamics of HIV in bar setting in slums would better characterise the HIV risk among individuals found at bars. Additionally, an evaluation of the prevalence of other sexually transmitted diseases (STDs) among individuals at bars in slums would be desirable since STDs increase the risk of HIV infection^49^.

Studies that have evaluated the prevalence of TB in bar settings have been contact investigations of an index case and their findings may not compare to our cross sectional evaluation of the TB prevalence. Nonetheless, using DNA fingerprinting, these studies demonstrate that bars are an avenue for TB transmission and could propagate an outbreak among customers, employees and members of their households^15,50–53^. Similar to our finding, Godoy et al.(2017), found that the prevalence of active TB was 16 per 1,000 population upon investigation of contacts of an index case (a bar staff)^17^. Conversely, Kline, Hedemark and Davies (1996) found it at 144 per 1,000 population among contacts of an index bar patron, including customers and employees^19^. The prevalence of TB among individuals found at bars in slum settlements is not well reported elsewhere using a cross sectional design other than contact tracing investigations.

Our results underscore the epidemiological and clinical interrelatedness of TB and HIV among individuals found at bars in three ways. First, history of TB treatment was associated with HIV and this could be attributed to HIV-related depletion of CD4 T–lymphocytes which are crucial for immune responses against TB^54^. Secondly, from our multivariable logistic regression model, the odds ratios of HIV infection increased with a higher CAGE score. This suggests a dose dependent relationship between HIV and problematic alcohol use that leads to high sexual risk behaviour and inconsistent condom use^55^. The association of a higher risk of HIV with higher CAGE scores is also reported by a meta-analysis of studies across Africa^56^. This dose dependant relationship has been observed between tuberculosis risk and alcohol use as well^22^. Lastly, cigarette smoking was associated with HIV infection in our study yet it is an established risk factor for tuberculosis^57^. Therefore, these results suggest that people living with HIV found at bars also had TB risk factors. This calls for concurrent screening for HIV and TB by national HIV and TB programs among high risk populations in slums^58^. We did not directly evaluate the association between HIV and TB in the multivariable model population due to the small number of TB outcomes. However, current TB treatment was commoner among HIV-positive individuals than HIV-negative persons (*p* = 0.035). We have not found reports that have concurrently evaluated the prevalence HIV and tuberculosis among individuals in bars in slum settings and thus our findings fill an important knowledge gap.

Our study had some limitations. We did not perform sputum cultures which could have increased our detection of bacteriologically confirmed tuberculosis. Further, by excluding participants with alcohol intoxication, we might have inadvertently excluded individuals with a higher risk for TB and HIV. However, this was necessary to ensure that consent to participate was obtained wilfully. Lastly, the small sample size affected the precision of the effect sizes of predictors of HIV infection. This analysis did not account for other confounders such as sexual behaviour, knowledge and practices regarding HIV and TB prevention and ease of access to HIV and TB diagnostic services, all of which could influence the risk of TB and HIV in this population. Subsequent studies could evaluate these factors among individuals found at bars with a larger sample size.

## Conclusion

There was a high prevalence of HIV and TB among individuals found at bars in the slums of Kampala, Uganda. Female sex, current cigarette smoking, history of TB treatment, and CAGE scores of 2–3 and 4 predicted HIV infection. These study findings highlight the need for concurrent programmatic screening for HIV and TB among high risk populations in slums to identify the missed infections that drive the two epidemics. We recommend slum specific programs for HIV and TB prevention that focus on women and reduction of cigarette smoking and alcohol use among individuals at bars in slum settings.

## Data Availability

Datasets generated and/or analysed during this study are available from the corresponding author upon reasonable request.
